# Learning the Language of Histopathology Images reveals Prognostic Subgroups in Invasive Lung Adenocarcinoma Patients

**DOI:** 10.21203/rs.3.rs-8089525/v1

**Published:** 2026-01-16

**Authors:** Abdul Rehman Akbar, Usama Sajjad, Ziyu Su, Wencheng Li, Fei Xing, Jimmy Ruiz, Wei Chen, Muhammad Khalid Khan Niazi

**Affiliations:** 1 Department of Pathology, College of Medicine, The Ohio State University Wexner Medical Center, Columbus, OH, USA; 2 Department of Pathology, Wake Forest University School of Medicine, Winston-Salem, NC, USA; 3 Department of Cancer Biology, Wake Forest University School of Medicine, Winston-Salem, NC, USA; 4 Department of Medicine (Hematology & Oncology), Wake Forest University School of Medicine, Winston-Salem, NC, USA; 5 Section of Hematology & Oncology, W.G. (Bill) Hefner Veterans Affair Medial Center (VAMC), Salisbury, NC, USA

## Abstract

Recurrence remains a major clinical challenge in surgically resected invasive lung adenocarcinoma, where existing grading and staging systems fail to capture the cellular complexity that underlies tumor aggressiveness. We present **PathRosetta**, a novel AI model that conceptualizes histopathology as a language, where cells serve as words, spatial neighborhoods form syntactic structures, and tissue architecture composes sentences. By learning this language of histopathology, PathRosetta predicts five-year recurrence directly from hematoxylin-and-eosin (H&E) slides, treating them as documents representing the state of the disease. In a multi-cohort dataset of **289 patients** (**600 slides**), PathRosetta achieved an area under the curve (AUC) of **0.78±0.04** on the internal cohort, significantly outperforming IASLC grading (AUC:0.71), AJCC staging (AUC:0.64), and other state-of-the-art AI models (AUC:0.62–0.67). It yielded a **hazard ratio of 9.54** and a **concordance index of 0.70**, generalized robustly to external TCGA (AUC:0.75) and CPTAC (AUC:0.76) cohorts, and performed consistently across demographic and clinical subgroups. Beyond whole-slide prediction, PathRosetta uncovered **prognostic subgroups within individual cell types**, revealing that even within benign epithelial, stromal, or other cells, distinct morpho-spatial phenotypes correspond to divergent outcomes. Moreover, because the model explicitly understands what it is looking at, including cell types, cellular neighborhoods, and higher-order tissue morphology, it is inherently interpretable and can articulate the rationale behind its predictions. These findings establish that representing histopathology as a language enables interpretable and generalizable prognostication from routine histology.

## Introduction

Lung adenocarcinoma, the most common histological subtype of non-small cell lung cancer (NSCLC), remains a leading cause of cancer-related death worldwide, accounting for over 40% of NSCLC cases and contributing to more than 1.7 million deaths annually [1, 2]. Among these, the majority (~70%) are invasive lung adenocarcinoma (ILA), which represent the focus of this study. Despite advances in early detection and surgical management, patient outcomes remain poor. Five-year survival rates range from 70–90% for stage I disease to below 10% for stage IV, reflecting the strong stage dependence of prognosis [3, 4]. However, even among patients with early-stage disease treated with curative-intent resection, 20–30% experience recurrence within five years, and this rate exceeds 50% in stage III [5]. These statistics underscore the urgent need for reliable prognostic tools to identify high-risk patients and guide postoperative management [6].

However, current prognostic tools offer alarmingly limited predictive value. The tumor node metastasis (TNM) staging system, considered the gold standard for treatment decisions, achieves only modest prognostic performance for overall survival prediction [7–9]. Even more concerning, studies specifically evaluating recurrence prediction show that TNM staging achieves an area under the curve (AUC) of merely 0.561, while tumor grading performs only marginally better at 0.573 [10]. Histologic subtyping, despite recent advances in the International Association for the Study of Lung Cancer (IASLC) grading system, demonstrates AUCs ranging from 0.68–0.70 for recurrence-free survival [11]. These disappointing results stem from inherent limitations including interobserver variability and inability of these coarse-grained systems to capture the complex biological heterogeneity of tumors [12, 13]. There is thus an urgent need for more robust, biologically informed histopathological signals that can capture the complex cellular and architectural features underlying tumor heterogeneity, which when integrated with molecular and clinical data, could enable the development of comprehensive prognostic tools with superior predictive accuracy.

The advent of digital pathology has revolutionized cancer research by enabling computational analysis of whole slide images (WSIs) of hematoxylin and eosin (H&E)-stained tissue sections, which contain rich information about tumor architecture, cellular morphology, and the tumor microenvironment (TME) that may harbor high-dimensional prognostic signals invisible to the naked eye [14, 15]. However, extracting meaningful signals from these gigapixel-sized images remains a significant challenge.

**Figure 1 |:**
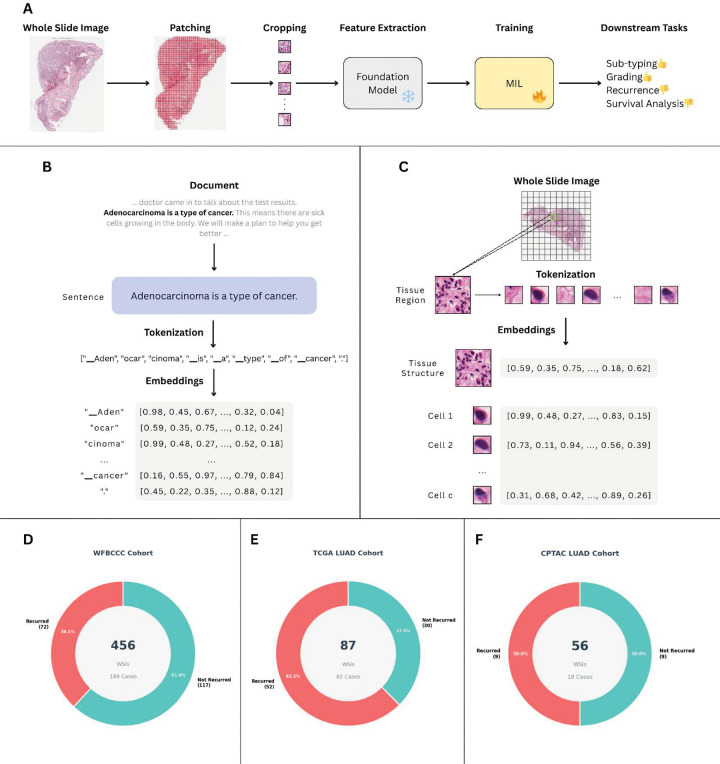
Conceptual overview of PathRosetta and study cohorts **(A)** Traditional computational pathology workflows treat a WSI as a set of patches that are cropped, processed through a foundation model, and aggregated using MIL for downstream tasks such as subtyping, grading, recurrence prediction, or survival analysis. **(B)** In natural language processing, meaning emerges from word arrangement and context. Language models encode this structure by tokenizing text and representing words as contextual embeddings that capture semantics and syntax. **(C)** PathRosetta applies this linguistic principle to histopathology by tokenizing tissue into individual cells, learning their morphological and spatial embeddings, and modeling cell–cell interactions as biological “sentences” that encode tumor behavior. **(D-F)** Cohorts used for training and validation: (D) internal WFBCCC cohort (456 WSIs, 189 cases, 38.1% recurrence); (E) TCGA-LUAD external test set (87 WSIs, 82 cases, 42.7% recurrence); and (F) CPTAC-LUAD external test set (56 WSIs, 18 cases, 50% recurrence). Together, these datasets enabled development and cross-institutional validation of PathRosetta as a framework that models the language of pathology.

The application of artificial intelligence (AI) in pathology has shown significant potential, not only for diagnosing tumors but also for predicting diverse clinical endpoints [15–24]. However, the majority of these AI approaches rely on foundation models (FMs), task-agnostic architectures trained on massive datasets in a self-supervised manner [25–29]. Natural images, the primary domain for which these paradigms were developed, lack inherent hierarchical structure. A photograph of a landscape or street scene contains pixels and regions, but these do not naturally organize into semantically defined building blocks. Modern vision transformers encode such images by dividing them into patches and learning relationships through self-attention, treating each patch as a token. Self-supervised methods such as DINO (Self-Distillation with No labels) [30] further refine this approach by encouraging similarity between different views of the same image. While these paradigms have demonstrated impressive success in general computer vision, their direct transfer to histopathology remains challenging. These limitations hinder FM performance on prognostic tasks such as survival and recurrence risk prediction, particularly when used as frozen feature extractors (Fig. 1A) [31].

In stark contrast, natural language processing has been revolutionized by an architectural insight: language possesses inherent hierarchical building blocks. Alphabets combine to form words, words assemble into phrases, phrases construct sentences, and so forth. Language models explicitly encode this structure, capturing meaning at multiple scales simultaneously (Fig. 1B). This hierarchical encoding, where the meaning of each element is determined by its context and position within the broader structure, has enabled unprecedented success in specialized domains.

Remarkably, histopathology represents a profound exception among visual domains: it possesses intrinsic biological building blocks analogous to linguistic structures. Individual cells serve as fundamental “words,” cellular neighborhoods function as “phrases,” tissue regions act as “sentences,” and whole slides constitute complete “documents.” Just as the meaning of a word shifts based on context, the biological significance of a cell is determined by its spatial neighbors and microenvironment. Despite this compelling analogy, computational pathology remains largely anchored to vision models designed for natural images, fundamentally overlooking the structured, hierarchical nature of biological tissues.

To fill this gap, we introduce PathRosetta, an AI framework that models the “language of histopathology” by explicitly treating tissue as a structured biological text. To the best of our knowledge, this represents the first study to explicitly leverage hierarchical, language-inspired modeling principles for computational pathology. In PathRosetta, individual cells act as fundamental tokens, their interactions and spatial neighborhoods form phrases, and higher-order tissue regions assemble into sentences, collectively defining the grammar of disease (Fig. 1C). By learning how cells “communicate” through their spatial and morphological context, PathRosetta captures the cellular syntax and semantic relationships that govern tumor architecture and microenvironmental organization. This biologically grounded representation enables robust outcome prediction and offers interpretable insights into the cellular and structural dynamics that shape disease behavior.

## Results

### PathRosetta outperforms clinical and computational benchmarks

**Figure 2 |:**
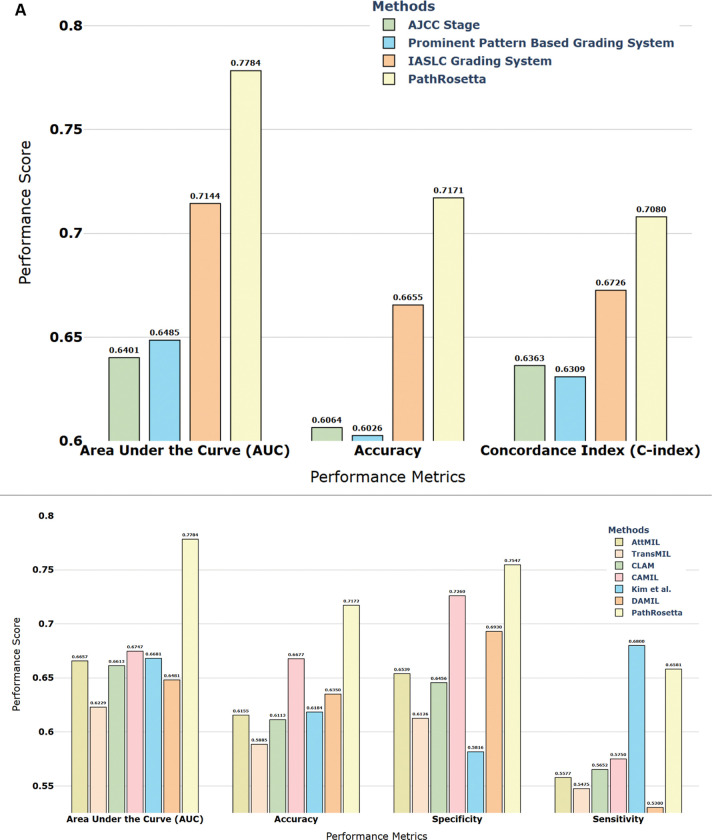
PathRosetta comparison with clinical and computational benchmarks **(A)** Comparison of PathRosetta with established clinicopathologic prognostic systems, including AJCC stage, the prominent pattern–based grading system, and the IASLC grading system. **(B)** Comparison with leading AI models, including AttMIL, TransMIL, CLAM, CAMIL, DAMIL, and Kim et al.

We evaluated PathRosetta for predicting 5-year recurrence in a cohort of 189 patients with ILA, using 456 H&E-stained WSIs (Fig 1D). Under a rigorous five-fold patient-wise cross-validation framework, PathRosetta substantially outperformed both established clinicopathologic variables and state-of-the-art AI models. Traditional baselines, including AJCC stage, dominant pattern–based grading, and the IASLC grading system, demonstrated limited prognostic power, with AUCs ranging from 0.64 to 0.71 and concordance indices of 0.63–0.67 (Fig. 2A). Similarly, leading AI models achieved a maximum AUC of just 0.67. In contrast, PathRosetta achieved an AUC of 0.78, representing a greater than 16% relative improvement over second-best AI method (AUC: 0.67) along with an accuracy of 71.7%, a sensitivity of 65.8%, and a specificity of 75.5% (Fig. 2B, Supplementary Table S2).

### Cell-type–specific language modeling reveals prognostically distinct subgroups within pathologically defined cell types

**Figure 3 |:**
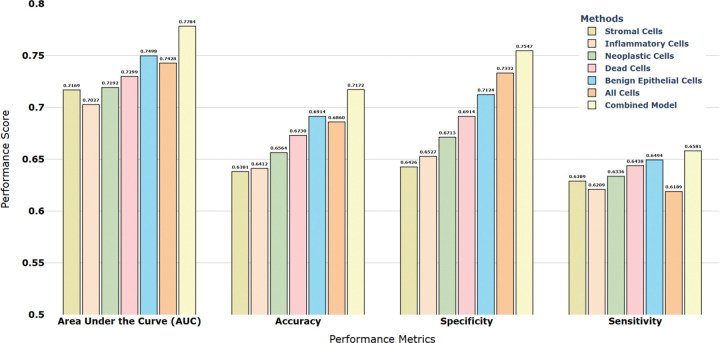
Cell-type–specific and ensemble model performance Performance comparison of PathRosetta models trained on individual cell types, the all-cell model, and the combined ensemble model for 5-year recurrence prediction in ILA. Each bar represents the mean performance across five cross-validation folds for four key metrics: area under the curve (AUC), accuracy, specificity, and sensitivity.

PathRosetta’s flexible architecture enables the construction of tissue “documents” from different cellular vocabularies. By treating each cell type as a distinct lexicon, we can generate separate histopathology languages, one composed solely of stromal cell “words,” another from inflammatory cells, and so forth. To investigate which cellular vocabularies carry the most prognostic information for recurrence prediction, we trained separate models using five cell-type–specific languages: stromal, inflammatory, neoplastic, benign epithelial, and dead cells. Each demonstrated distinct yet complementary prognostic value revealing subgroups within a cell type with divergent outcomes. Among these, the benign epithelial cell model performed best (AUC = 0.75), followed by dead cells (AUC = 0.73) and stromal cells (AUC = 0.72), while inflammatory and neoplastic cell models also showed solid discriminative ability (AUCs = 0.70–0.72). An All-Cell model, trained on pooled cellular features without distinguishing types, achieved an AUC of 0.74, while the combined ensemble model, integrating predictions across all cell-type–specific models, achieved the highest performance (AUC = 0.78, accuracy = 71.7%, specificity = 75.5%) (Fig. 3, Supplementary Table S3).

### Risk stratification and survival analysis

**Figure 4 |:**
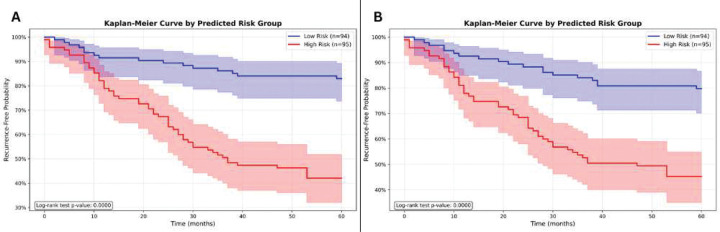
Kaplan–Meier survival analysis based on predicted recurrence risk Kaplan–Meier recurrence-free survival curves for patients stratified into high- and low-risk groups based on model predictions. **(A)** The All-Cells model trained using pooled cellular features without distinguishing between cell types. **(B)** The Combined ensemble model, which integrates predictions from all cell-type–specific models.

We next assessed clinical utility using Cox proportional hazards regression. PathRosetta achieved the strongest risk stratification, with a hazard ratio (HR) of 9.54 (95% CI: 4.34–20.98, p < 0.005), indicating a more than ninefold higher recurrence risk for patients predicted as high-risk compared to low-risk. The All-Cell model also demonstrated substantial prognostic separation (HR = 7.70, 95% CI: 3.80–15.60, p < 0.005) (Supplementary Table S4). Kaplan–Meier analyses confirmed clear and statistically significant separations between high- and low-risk groups across all models (p < 0.0001; Fig. 4). The benign epithelial and dead cell models achieved the strongest stratification among individual cell types (HRs = 6.04 and 5.69, respectively), consistent with their superior AUC and concordance index values (Supplementary Table S4).

### PathRosetta demonstrates consistent performance across demographic and clinical subgroups

**Figure 5 |:**
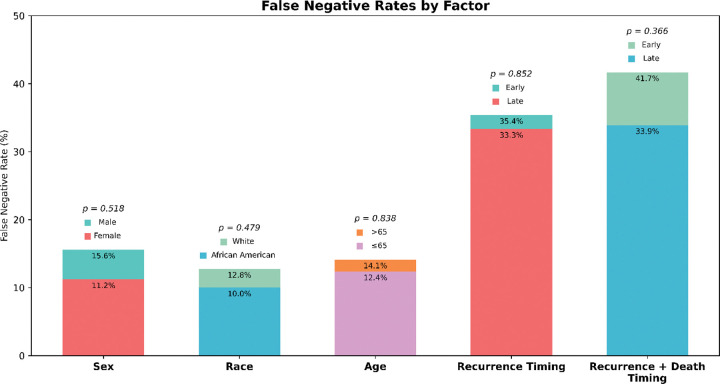
Evaluation of model fairness and failure patterns across demographic and clinical subgroups False negative rates for the PathRosetta were compared across demographic (sex, race, age) and clinical (recurrence timing, recurrence + death timing) subgroups. Bars represent mean false negative rates within each group, and p-values indicate statistical test results for group differences.

To ensure fairness and robustness, we analyzed PathRosetta’s performance across demographic and clinical subgroups, including sex, race, age, tumor stage, and histologic grade. No significant differences in false negative rates were observed across sex (female = 11.2%, male = 15.6%; p = 0.52), race (White = 12.8%, African American = 10.0%; p = 0.48), or age groups (≥65 years = 14.1%, <65 years = 12.4%; p = 0.84). There was also no statistically significant difference between recurrence and death timings of the patients (Fig. 5). Performance trends followed clinical expectations, with higher AUCs in well-differentiated (IASLC Grade 1, AUC = 0.87) and early-stage (Stage I, AUC = 0.75) tumors, and lower scores in advanced disease (Stage III, AUC = 0.53). The diminished accuracy in Stage III cases likely reflects small sample size (n = 15) and uneven fold representation (Supplementary Table S5). These analyses were done on the respective test sets of each fold’s trained model.

### External validation confirms generalizability

**Figure 6 |:**
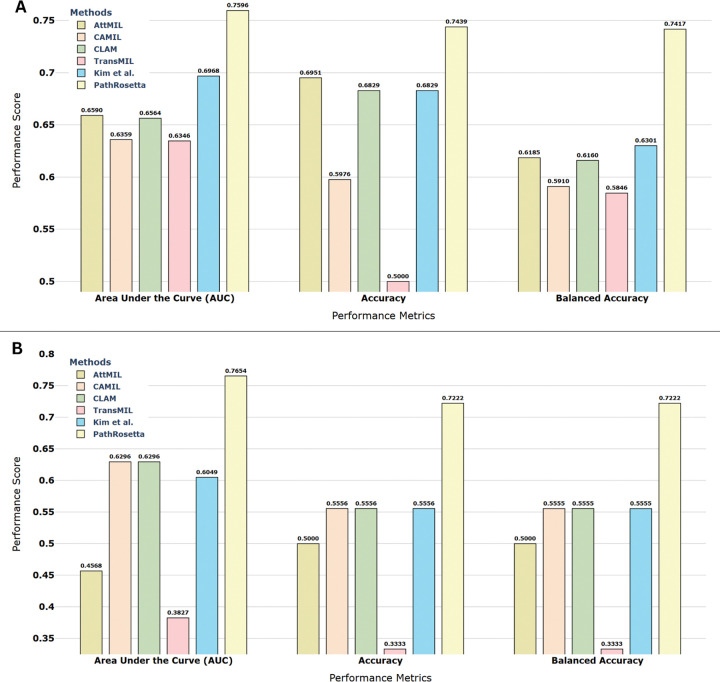
External validation of PathRosetta on independent LUAD cohorts Performance comparison of PathRosetta with leading AI models on two independent datasets. **(A)** Results on the TCGA-LUAD cohort (82 cases, 87 WSIs) showing that PathRosetta achieves the highest performance across all metrics, outperforming all comparative methods, including AttMIL, CAMIL, CLAM, TransMIL, and Kim et al. **(B)** Results on the CPTAC-LUAD cohort (18 cases, 56 WSIs), where PathRosetta again demonstrates superior generalization despite variations in slide preparation and scanning protocols.

To evaluate the generalizability of PathRosetta beyond the development cohort, we tested the model on two independent external datasets: the TCGA-LUAD (Fig. 1E) and CPTAC-LUAD (Fig. 1F) cohorts. Despite variations in slide preparation, staining, and scanning protocols, PathRosetta maintained strong predictive performance, achieving AUCs of 0.75 and 0.76 on TCGA and CPTAC, respectively. The model also demonstrated balanced accuracy and specificity exceeding 72% across both datasets, indicating robust discrimination between patients with and without recurrence (Fig. 6). Detailed quantitative comparisons with other methods are provided in Supplementary Table S6.

## Discussion

Traditional histopathology models treat WSIs as collections of patches, overlooking the basic biological reality that cancer is a cellular disease [32]. Tumors are composed of heterogeneous cell populations that differ subtly in morphology, molecular state, and function. Prior studies have shown that features such as nuclear shape, texture, and size, often imperceptible to the human eye, carry strong prognostic value [33, 34]. Moreover, TME includes dynamic networks of stromal, immune, and epithelial cells that modulate disease progression and treatment response. Together, these findings highlight that accurate outcome prediction requires modeling not only the presence of specific cells, but also their spatial context and interactions.

PathRosetta operationalizes this concept by treating individual cells as the basic “tokens” of a histopathological language. Analogous to how language models derive meaning from the arrangement of words, PathRosetta learns how spatial and morphological relationships among cells form the biological “sentences” that encode the state of the tumor ecosystem. The model captures not only what cells exist, but the context in which they coexist, information that patch-based methods inherently dilute.

**Figure 7 |:**
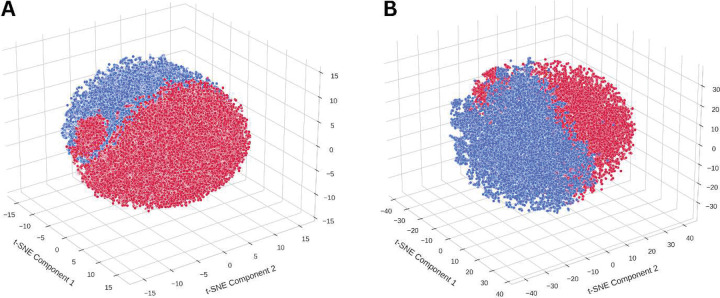
Visualization of sub-visual cellular heterogeneity in benign epithelial cells Three-dimensional t-SNE projections of cell-level embeddings extracted using CellViT++ for benign epithelial cells from recurrence-positive (red) and recurrence-negative (blue) patients in our internal cohort. **(A)** and **(B)** represent two independently sampled subsets of the same cohort, illustrating consistent clustering patterns across experiments. In both instances, cells from recurred and not recurred patients occupy distinct regions of the embedding space, despite being histologically indistinguishable.

To illustrate this principle, we analyzed cell-level embeddings extracted using CellViT++ [35, 36] for five major cell categories. Visualization of cell embeddings revealed subgroups within benign epithelial cells of patients with and without recurrence, even though the cells appeared histologically similar (Fig. 7, Supplementary Fig. S1). This visual separation within the subgroups of same cell type reflects hidden molecular or functional states that influence disease behavior, emphasizing that cancer is not merely a disorder of morphology but a complex system of cellular states and communications. By operating in high-dimensional feature spaces, PathRosetta captures this latent heterogeneity, detecting subtle, prognostically relevant differences invisible to naked eye.

Building on this foundation, PathRosetta achieved substantial performance gains over state-of-the-art AI models and clinical systems. While traditional models treat patches as atomic units, averaging away cellular detail, PathRosetta explicitly preserves cell identity, morphology, and spatial arrangement. This enables the model to learn higher-order phenomena such as tumor–immune interplay, desmoplastic remodeling, and necrosis, which are critical determinants of progression and recurrence. This is quantitatively validated in our results where we showed that the PathRosetta outperformed cell-only and patch-only baselines by large margins (AUC: 0.78 vs. 0.64 and 0.67), demonstrating that recurrence risk is determined not solely by cell presence, but by how these cells interact and are spatially arranged (Supplementary Table S1).

The clinical significance of these improvements is reflected in the model’s ability to stratify patients into biologically distinct risk groups. PathRosetta achieved a hazard ratio of 9.54 between predicted high- and low-risk patients, suggesting strong utility for guiding adjuvant therapy and surveillance strategies in early-stage ILA. External validation on independent TCGA and CPTAC LUAD cohorts (AUCs 0.75 and 0.76) further confirms the model’s robustness and generalizability across data sources and staining variations.

A key strength of PathRosetta lies in its flexibility that it can construct prognostic “documents” from either the complete cellular ecosystem or from individual cell-type populations. Because it treats each cell as a fundamental unit (analogous to a word in language) and builds hierarchical representations through their spatial relationships, the model allows selective interrogation of which cellular compartments encode the most informative prognostic signals. This capability is intrinsic to our language-inspired architecture, enabling systematic evaluation of the prognostic contribution of each cell type independently while preserving the hierarchical language of pathology at both cellular and spatial scales. Importantly, the cell-type–specific analyses revealed subgroups within different cell types which offer complementary prognostic signals. When we constructed a “document” composed solely of benign epithelial cells and defined its grammar using PathRosetta, this model achieved the highest performance (AUC = 0.75), indicating that non-neoplastic epithelial morphology and organization carry underappreciated prognostic information, potentially reflecting early field effects or adaptive epithelial responses. These results extend the traditional focus on malignant cells alone, reinforcing that cancer progression emerges from the collective dynamics of the tumor ecosystem. To definitively link these computational subgroups to recurrence, we will perform spatial transcriptomics to resolve the specific gene expression programs and molecular differences that drive risk within the pathologically defined cell type.

Interpretability analyses support these biological insights. High-risk predictions consistently localized to regions with solid and micropapillary architecture, nuclear atypia, and necrosis, while low-risk predictions focused on immune-rich or fibrotic areas associated with favorable outcomes. These attention patterns align with established histologic correlates of prognosis, underscoring that PathRosetta’s representations are both data-driven and biologically grounded. Representative attention maps for high- and low-risk patients are shown in Supplementary Figures S2 and S3.

Collectively, these findings highlight a conceptual shift in computational pathology, from image-level recognition to language modeling. By explicitly representing tissue as a network of interacting cells, PathRosetta decodes the hierarchical grammar that underlies tumor behavior. This biologically informed design establishes a scalable framework for generalization across cancer types, endpoints, and modalities. Future extensions integrating molecular and spatial transcriptomic data may further connect cellular morphology to gene expression and functional state, deepening our understanding of how tissue organization encodes clinical outcomes.

In summary, PathRosetta reframes histopathology as a decipherable biological language, one in which each cell contributes meaning through its morphology, neighbors, and spatial context. By learning this language, PathRosetta not only advances prognostic modeling but also provides a foundation for interpretable, biologically faithful AI systems in precision oncology.

## Methods

### Study design and patient cohorts

This study utilized a cohort of 189 patients diagnosed with stage I to III ILA, who underwent curative-intent surgical resection at Wake Forest Baptist Comprehensive Cancer Center between 2008 to 2015. All patients were followed up for a minimum of five years. The cohort included 456 H&E-stained WSIs, with a median of two slides per patient. All slides corresponding to a given patient were assigned to the same fold to avoid data leakage. Patient follow-up data included recurrence status and time to recurrence, with 72 patients (38.1%) experiencing recurrence within five years. The study design diagram (Supplementary Fig. S4A) outlines the exclusion criteria and study population, while cohort demographics (Supplementary Fig. S4B) show a balanced distribution of age, with slightly more females (56.6%) than males (40.7%), and the sex of 5 patients (2.6%) unknown. Notably, patients who experienced recurrence had higher rates of lymphovascular invasion (29.2% vs. 7.7%) and higher-grade tumors (84.7% vs. 43.6% for Grade 3 in the new grading system, WHO 5th Ed. [2021]). For external validation, we used two independent public datasets: the TCGA-LUAD (82 cases) and CPTAC-LUAD (18 cases) cohorts. Only cases with available 5-year recurrence data were included.

### PathRosetta Architecture

**Figure 8 |:**
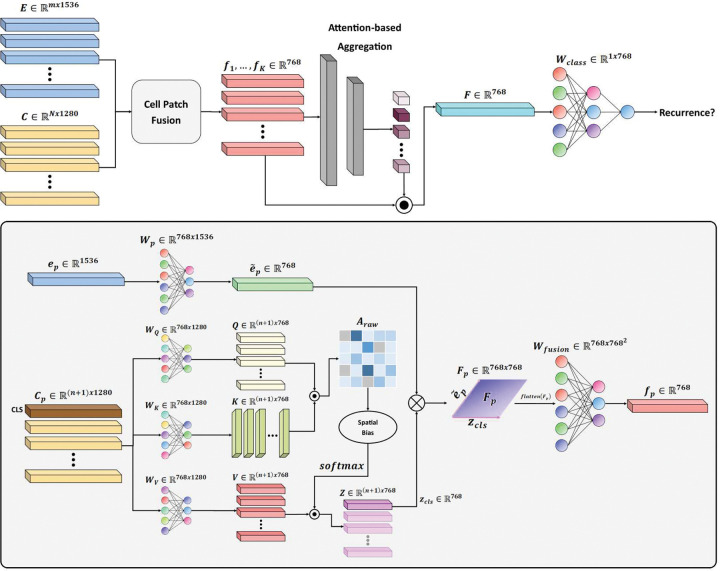
Schematic overview of the PathRosetta architecture Top: PathRosetta integrates patch-level and cell-level representations through a cell–patch fusion mechanism, followed by attention-based aggregation for whole-slide recurrence prediction. Patch and cell embeddings are projected into a shared latent space and fused to form unified representations which are subsequently aggregated into a slide-level feature vector for recurrence classification. **Bottom:** Detailed architecture of the fusion module. Cell embeddings are combined using a multi-head self-attention mechanism with spatial bias and fused with patch embeddings using outer product. The resulting fused feature map is flattened and passed through a learned fusion layer to generate the final fused representation. This hierarchical design enables PathRosetta to jointly capture cellular morphology, spatial context, and tissue architecture.

PathRosetta implements the “language of histopathology” concept through three core components that mirror natural language processing architectures: (1) multi-scale feature extraction captures both “words” (individual cells at 40×) and “sentences” (tissue patches at 20×), (2) cell-to-patch mapping and fusion creates contextual embeddings by linking cellular “words” with their architectural “sentences,” and (3) multi-dimensional attention mechanisms learn complex semantic relationships between tissue regions, analogous to how transformers capture dependencies between sentence elements. This architecture addresses the fundamental limitations of current AI methods by integrating information across multiple scales and explicitly modeling cellular and intercellular characteristics within their biological context. Figure 8 illustrates the overall architecture of PathRosetta.

#### Multi-Scale Feature Extraction

Our multi-scale feature extraction strategy implements the core principle of the pathology language: cells at 40× magnification serve as the fundamental “words” encoding fine-grained morphological and phenotypic information, while tissue patches at 20× magnification represent “sentences” that provide architectural context and spatial organization. This dual-scale approach mirrors how language models require both token-level understanding (individual word meanings) and sequence-level comprehension (sentence structure and context) to achieve semantic understanding. Just as word embeddings capture lexical information while sentence embeddings capture syntactic and semantic relationships, our cellular embeddings encode morphological details while patch embeddings capture tissue architecture and spatial patterns.

We adopted a two-step feature extraction strategy:

- **Patch-Level Feature Extraction (20x):** WSIs were tiled into non-overlapping 256×256 pixel patches at 20x magnification. Using a pre-trained FM, UNI2-h [25] , we extracted a feature vector $e_{p}\in R^{1536}$ for each patch p, capturing broad morphological and tissue-architectural patterns. We used Trident [37, 38] for tissue segmentation, patch cropping, and feature extraction.
- **Cell-Level Feature Extraction (40x):** Concurrently, cell nuclei were segmented and classified from WSIs at 40× magnification using CellViT++ [35, 36], specifically the SAM-based model [39]. This model yields embeddings $c_{i}\in R^{1280}$ for each individual cell $c_{i}$, encoding fine-grained morphological and phenotypic details across five major cell categories: stromal, inflammatory, neoplastic, dead, and benign epithelial. It also outputs centroid coordinates for each cell. Preprocessing was performed using PathoPatcher [40].

This dual-scale approach (20x for summarizing path level information and 40x for capturing subtle information at cell level) overcomes the limitations of single-scale modeling, enabling the simultaneous utilization of both cellular morphology and tissue architecture.

#### Cell-to-Patch Mapping and Spatially Biased Cell Attention

The integration of cellular and tissue-level information implements contextual embedding principles from natural language processing, where the meaning of individual elements depends critically on their surrounding context. In PathRosetta, a cell’s biological significance, like a word’s semantic meaning, is determined not only by its intrinsic morphological features but also by its spatial neighbors and architectural context. The spatially biased attention mechanism we introduce mimics how attention mechanisms in language models allow words to attend to relevant context, enabling our framework to automatically learn which cellular neighborhoods are most informative for recurrence prediction.

#### Cell-to-Patch Mapping

We established a spatial correspondence between cells and patches by mapping each cell to the patch containing its centroid. Formally, for each patch p, we define the set of corresponding cells as:

$$C_{p}=\left\{c_{i}\in C|\mathrm{centroid}\left(c_{i}\right)\in p\right\},$$

where $C_{p}$ is the set of cells whose centroids lie within the spatial bounds of patch p, and C is the full set of cells. This mapping preserves spatial context, linking cells to the patch that contains them.

#### Spatially Biased Cell Self-Attention

Each patch embedding $e_{p}\in R^{1536}$, was linearly projected to $R^{768}$ via a learned projection:

$${\tilde{e}}_{p}=W_{p}e_{p},\;\mathrm{where}\;W_{p}\in R^{768\times 1536}$$

Each patch p contains c cells, we denote the cell embeddings as $c_{1},c_{2},c_{3},\ldots ,c_{c}\in R^{1280}$. Each $c_{i}$ is passed through LayerNorm both before and after attention. We aggregate these variable-length cell embeddings using a self-attention mechanism with a learnable CLS token $e_{CLS}\in R^{1280}$. The input sequence is:

$$C_{p}=\left[e_{CLS},c_{1},\ldots ,c_{c}\right]\in R^{(n+1)\times 1280}$$

We apply learned projections:

$$Q=W_{Q}\cdot X,\;K=W_{K}\cdot X,\;V=W_{V}\cdot X,$$

where $W_{Q},\;W_{K},\;W_{V}\in R^{768\times 1280}$.

We computed the raw attention scores between all elements:

$$A_{raw}=\frac{\left(Q\;\cdot \;K^{T}\right)}{\sqrt{d_{K}}},$$

where $d_{K}$ is the dimension of the key vectors i.e., 768. Then, we incorporated spatial information by subtracting the physical distance between cell centroids from the attention scores:

$$A_{\mathrm{spatial}(i,j)}=A_{\mathrm{raw}(i,j)}-dist\left({\mathrm{centroid}}_{i},{\mathrm{centroid}}_{j}\right),$$

where dist(⋅,⋅) is the Euclidean distance between cell centroids. This spatial bias implements the grammatical rules of pathology language, where cellular proximity defines semantic relationships—just as word order and adjacency create meaning in natural language, spatial arrangements of cells encode biological function and prognostic significance. Cells in close proximity form functional neighborhoods that act as coherent semantic units within the tissue microenvironment. This enables the framework to capture local cellular neighborhoods and their collective behavior, which is crucial for understanding TME characteristics and their role in cancer progression. We then applied softmax normalization to obtain the final attention weights:

$$A=softmax\left(A_{\mathrm{spatial}}\right)$$

The output of the self-attention layer is computed by:

Z=A⋅V

The updated CLS token $z_{CLS}\in R^{768}$ is extracted from the first row of Z, which now contains aggregated information from all cells within the patch, with greater influence from spatially proximal cells.

This spatially biased self-attention mechanism allows the framework to selectively focus on informative cells while capturing cell-cell interactions within each patch, addressing the homogeneous treatment of cells in current approaches.

#### Cross-Scale Fusion

Cross-scale fusion represents the critical step where “word-level” cellular information is integrated with “sentence-level” tissue architecture, analogous to how transformer models combine token embeddings with positional and contextual information. This fusion captures the biological reality that cellular behavior cannot be understood in isolation—just as word meaning depends on sentence context, cellular significance emerges from the interplay between intrinsic cell properties and their tissue microenvironment. The outer product operation we employ creates a rich representation space that captures all possible interactions between cellular morphology and architectural context.

For each patch p, we have a patch embedding vector ${\tilde{e}}_{p}\in R^{768}$ and an aggregated cell embedding vector $z_{\mathrm{CLS}}\in R^{768}$, we computed the outer product between these vectors:

$$F_{p}={\tilde{e}}_{p}⊗z_{CLS}\in R^{768\times 768}$$

This matrix captures all pairwise multiplicative interactions between patch and cell features. To make this representation computationally tractable, we flattened the matrix and projected it back to 768 dimensions using a learnable projection matrix $W_{\mathrm{fusion}}$:

$$f_{p}=W_{\mathrm{fusion}}\cdot f\;\mathrm{latten}\;\left(F_{p}\right),\;W_{\mathrm{fusion}}\in R^{768\times 768^{2}}$$

where $f_{p}\in R^{768}$ is the final fused representation for patch p.

This fusion mechanism enables rich cross-scale interactions that would be missed by simpler concatenation or addition operations, allowing the framework to capture how cellular characteristics interact with tissue architecture to influence recurrence risk.

#### Multi-Dimensional Attention MIL

The fused embeddings $f_{1},\;\ldots \;,f_{K}\in R^{768}$ from a WSI form a “bag” of K instances. We employed AttMIL to aggregate these instance features into a slide-level representation for predicting recurrence. This concept is visualized in Figure 2(B).

#### Conventional 1D Attention (AttMIL)

As a baseline and part of our ablation, we used the standard AttMIL, where instance embeddings $h_{k}$ (our $f_{k}$) are projected onto a 1D space, see the equation below for reference, to get attention weights $\alpha_{k}$, which are then used to compute a weighted sum of instance embeddings.

$$\alpha_{k}=\frac{exp\;\{w^{T}\left(tanh\left(Vh_{k}^{T}\right)\;⊙\;sigmoid\left(Uh_{k}^{T}\right)\right)\}}{\sum_{j=1}^{K}exp\;\{w^{T}\left(tanh\left(Vh_{j}^{T}\right)\;⊙\;sigmoid\left(Uh_{j}^{T}\right)\right)\}}$$

where V,U are learnable subspaces, and ⊙ denotes element-wise multiplication. The scalar $w^{T}(\cdot )$ collapses the transformed instances into a single attention score. The reliance on a single weight vector w can produce identical attention scores for instances with divergent feature interactions when projected onto the 1D subspace defined by w.

#### Novel 2D and 3D Attention Projections

To overcome the limitations of 1D projection, we extended this by projecting $h_{k}$ onto a 2D or 3D plane/space using two $\left(w_{1},w_{2}\right)$ or three $\left(w_{1},w_{2},w_{3}\right)$ learnable weight vectors, respectively. This can be written mathematically as:

$$\alpha_{k}=\frac{exp\;\{{\left\|W^{T}\left(tanh\left(Vh_{k}^{T}\right)\;⊙\;sigmoid\left(Uh_{k}^{T}\right)\right)\right\|}_{F}\}}{\sum_{j=1}^{K}exp\;\{{\left\|W^{T}\left(tanh\left(Vh_{j}^{T}\right)\;⊙\;sigmoid\left(Uh_{j}^{T}\right)\right)\right\|}_{F}\}}$$

where $\|\cdot {\|}_{F}$ is the Frobenius norm. This allows the framework to assign importance based on a richer, multi-faceted representation of each instance.

#### Cell-Type Specific Models

Cell-type-specific modeling reflects the concept that different cellular populations represent distinct “vocabularies” within the pathology language, each with specialized semantic roles in the TME. Just as domain-specific language models excel in specialized contexts (medical texts, legal documents, scientific literature), our cell-type-specific models become experts in interpreting the unique contributions of different cellular populations to recurrence risk. The ensemble approach combines these specialized “linguistic perspectives,” recognizing that comprehensive understanding of tissue biology—like natural language comprehension—benefits from integrating multiple specialized knowledge domains.

Let the five major cell types be denoted by the set:

T={stromal,inflammatory,neoplastic,dead,benignepithelial}

#### Cell-Type Specific Subsets

For each cell type t∈T, we constructed a subset of each WSI as follows: Let $C_{t}\subseteq C$ be the set of all cells of type t present in a WSI.

Using the spatial mapping dictionary, we selected the set of patches $P_{t}\subseteq P$, where each patch $p\in P_{t}$ satisfies:

$$C_{p}\cap C_{t}\neq \emptyset ,$$

meaning the patch contains at least one cell of type t.

Each model $M_{t}$ was then trained using the subset $S_{t}=\left\{P_{t},C_{t}\right\}$, resulting in five specialized models focused on learning the recurrence-related patterns associated with a specific cell type.

A sixth model, denoted $M_{\text{all}}$, was trained using all cell embeddings and all patches that contained at least one cell of any type:

$$P_{\text{all}}=\left\{p\in P|C_{p}\neq \emptyset \right\}$$

This model captures holistic cellular heterogeneity and serves as a comprehensive multi-cell-type baseline.

#### Ensemble Integration via Patient-Level Majority Voting

To integrate predictions across cell-type-specific perspectives, we employed a patient-level ensemble approach based on majority voting.

Let a patient i have $S_{i}$ WSIs, and let $M=\left\{M_{1},M_{2},\;\ldots \;,M_{6}\right\}$ be the six trained models (five cell-type-specific and one multi-type model). Each model $M_{m}\in M$ outputs a probability score ${p_{i,s}}^{\left(m\right)}\in [0,\;1]$ for slide $s\in \left\{1,\;\ldots \;,S_{i}\right\}$ of patient i.

The aggregated patient-level probability for model $M_{m}$ is computed as the average over all WSIs:

$${\tilde{p}}_{i}^{\left(m\right)}=\frac{1}{S_{i}}\sum_{s=1}^{S_{i}}p_{i,s}^{\left(m\right)}$$

The final patient-level prediction is then obtained by averaging the probabilities across all models:

$${\tilde{p}}_{i}=\frac{1}{\left|M_{i}\right|}\sum_{m\in M_{i}}{\tilde{p}}_{i}^{\left(m\right)},$$

where $M_{i}\subseteq M$ includes only the models applicable to patient i (i.e., for which at least one relevant cell is found).

The final binary decision is made using a threshold τ∈[0,1](τ=0.5):

$${\overset{ˆ}{y}}_{i}=\left\{\begin{array}{ll} 1, & \;\mathrm{if}\;{\tilde{p}}_{i}\geq \tau \\ 0, & \mathrm{otherwise} \end{array}\right.$$

This ensemble strategy leverages the distinct predictive power of each cell type while combining them to improve generalizability and robustness. It also reflects the biological heterogeneity of the TME, where different cell populations contribute uniquely to tumor progression and recurrence.

### Training and optimization

Models were trained using five-fold cross-validation on NVIDIA A100 GPUs for up to 100 epochs with early stopping based on validation performance. Optimization was performed using the Adam optimizer with an initial learning rate of 5 × 10^−5^, weight decay of 1 × 10^−5^, and binary cross-entropy loss with L2 regularization. To ensure clinically balanced predictions, we introduced a harmonized stopping criterion that maximizes the harmonic mean of sensitivity and specificity on the validation set. The model corresponding to the best validation epoch was used for testing.

### Baseline and comparative methods

We compared PathRosetta against several state-of-the-art computational pathology methods, including AttMIL [41], CLAM [42], TransMIL [43], CAMIL [44], DAMIL [20], and Kim et al. (EfficientNet-b0 + AttMIL) [45]. Clinical baselines included AJCC stage, dominant pattern grading, and the IASLC grading system. For clinical systems, AUCs were computed using machine learning models like logistic regression, random forest, xgboost, support vector machine, etc. and C-indices from Cox proportional hazards models.

### Evaluation metrics

Performance was evaluated using AUC, accuracy, sensitivity, specificity, and C-index. Statistical significance was assessed using the log-rank test for Kaplan–Meier analyses and 95% confidence intervals for hazard ratios (Cox models).

All metrics represent mean ± standard deviation across test folds. External validation results on TCGA and CPTAC cohorts were computed on the model having the best performance on validation set and are reported separately (Supplementary Table S6).

### Statistical analysis

All statistical analyses were performed using Python 3.10 with scikit-learn 1.3, lifelines 0.27, and statsmodels 0.14. A p-value < 0.05 was considered statistically significant.

## Supplementary Material

Supplementary Files

This is a list of supplementary files associated with this preprint. Click to download.

• PathRosettanpjDigitalMedSupplementary.docx

## Data Availability

The data that support the findings of this study are available from Wake Forest Baptist Comprehensive Cancer Center, but restrictions apply to the availability of these data, which were used under license for the current study, and so are not publicly available. Data are, however, available from the authors upon reasonable request and with permission of Wake Forest Baptist Comprehensive Cancer Center. TCGA-LUAD is available through the National Cancer Institute’s Genomic Data Commons (GDC) portal (https://portal.gdc.cancer.gov) and CPTAC-LUAD is available through the Cancer Imaging Archive (TCIA) portal (https://www.cancerimagingarchive.net/collection/cptac-luad/).
